# IL-6 facilitates cross-talk between epithelial cells and tumor- associated macrophages in *Helicobacter pylori*-linked gastric carcinogenesis

**DOI:** 10.1016/j.neo.2024.100981

**Published:** 2024-02-28

**Authors:** Bingting Yu, Danny de Vos, Xiaopei Guo, SanFei Peng, Wenjie Xie, Maikel P. Peppelenbosch, Yang Fu, Gwenny M. Fuhler

**Affiliations:** a Department of Gastroenterology and Hepatology, Erasmus MC University Medical Center Rotterdam, Dr Molewaterplein 40, Rotterdam, GD 3015, the Netherlands; b Department of Gastrointestinal Surgery, The First Affiliated Hospital of Zhengzhou University, Zhengzhou, China; c Department of Infectious Diseases, Leiden University Medical Centre, the Netherlands; d Department of Parasitology, Leiden University Medical Centre, the Netherlands

**Keywords:** *Helicobacter pylori*, Interlukin-6, Gastric cancer, Cross-talk, Loop feedback, Microenvironment, M2 macrophage

## Abstract

**Purpose:**

*Helicobacter pylori (H. pylori)* is a significant risk factor for development of gastric cancer (GC), one of the deadliest malignancies in the world. However, the mechanism by which *H. pylori* induces gastric oncogenesis remains unclear. Here, we investigated the function of IL-6 in gastric oncogenesis and macrophage-epithelial cell interactions.

**Methods:**

We analyzed publicly available datasets to investigate the expression of *IL-6* and infiltration of M2 macrophages in GC tissues, and determine the inter-cellular communication in the context of IL-6. Human gastric epithelial and macrophage cell lines (GES-1 and THP-1-derived macrophages, respectively) were used in mono- and co-culture experiments to investigate autocrine-and paracrine induction of IL-6 expression in response to *H. pylori* or IL-6 stimulation.

**Results:**

We found that IL-6 is highly expressed in GC and modulates survival. M2 macrophage infiltration is predominant in GC and drives an IL-6 mediated communication with gastric epithelium cells. *In vitro*, IL-6 triggers its own expression in GES-1 and THP-1-derived macrophages cells. In addition, these cell lines are able to upregulate each other's IL-6 levels in an autocrine fashion, which is enhanced by *H. pylori* stimulation.

**Conclusion:**

This study indicates that IL-6 in the tumor microenvironment is essential for intercellular communication. We show that *H. pylori* enhances an IL-6-driven autocrine and paracrine positive feedback loop between macrophages and gastric epithelial cells, which may contribute to gastric carcinogenesis.

## Introduction

Despite a reduction in frequency over the past few decades, in the year 2020, gastric cancer (GC) remained the fifth most prevalent cause of cancer worldwide, affecting 1,089,103 people. In addition, GC remains the fourth most common cause of cancer-related mortality, accounting for 768,793 deaths worldwide. (Cancer today, *https://gco.iarc.fr/today/*). Infection of the gastric mucosa with the gram-negative bacterium *Helicobacter pylori* (*H. pylori*) is a key risk factor for GC development [1]. Chronic *H. pylori* infection can lead to atrophic gastritis, intestinal metaplasia (IM) and, ultimately, GC. The inflammatory environment created by chronic infection is thought to play a significant role in *H. pylori*-induced gastric carcinogenesis [2,3]. However, while *H. pylori* infects nearly 50% of the world's population, only a small percentage of these people develop GC [4]. The factors driving this discrepancy remain unclear.

A recent study showed that the extent of inflammation in the stomach following *H. pylori* eradication is greater in patients that continue to develop GC than in those who do not [5]. Multiple lines of evidence indicate that the pro-inflammatory cytokine interleukin-6 (IL-6) is a crucial factor in gastric carcinogenesis [6], [7], [8], [9], [10]. Previous studies showed a substantial and relevant association between the systemic IL-6 levels induced by *H. pylori*-infection and the relative incidence of GC [11]. IL-6 serum levels are increased in GC patients, and are associated with disease progression and worse prognosis [9,12]. In addition, polymorphisms in the genes encoding IL-6 and its cognate receptor have been identified as genetic risk factors for the development of GC [13]. Experimental evidence indicates that IL-6 enhances the proliferation [14] and invasiveness [15] of stomach cancer cell lines, and the overexpression of IL-6 in mice results in the development of multiple carcinomas [16], [17], [18]. In contrast, IL-6 knock-out mice presented a lower incidence of GC and reduced tumor size [19]. Taken together, these results suggest that an exaggerated IL-6 response contributes to the development of GC.

Signal transducer and activator of transcription-3 (STAT3) is phosphorylated during IL-6 signaling, allowing for its nuclear translocation and the activation of a number of downstream target genes. IL-6 itself is one of these target genes, and may thereby encourage its own synthesis, creating a positive feedback loop for IL-6 production. Examples of such a feedback loop are observed in the development of liver cancer [20] as well as the maintenance of myofibroblast activity in breast cancer [21]. Nonetheless, it remains unclear whether such a positive feedback loop also contributes to the development of chronic inflammation after *H. pylori* infection and stomach carcinogenesis.

Importantly, the IL-6/STAT3 axis is also involved in the polarization of macrophages, which are believed to be mediators in *H. pylori*-associated gastritis [22], through release of IL-6 [22,23]. The presence of macrophages, particularly those of the M2 type associated with immune evasion, is a characteristic of stomach carcinomas [24] and has been linked to the likelihood of disease development and mortality [25], [26], [27]. However, little is known regarding the role of IL-6 in the interplay between gastric epithelial cells and macrophages, and how this may drive gastric carcinogenesis.

Here, we investigated the role of IL-6 in gastric oncogenesis and the interaction between macrophages and epithelial cells. We demonstrate that IL-6 is strongly expressed in GC and promotes several tumor hallmarks. In addition, we show that IL-6 functions in a positive regulatory cross-talk between macrophages and epithelial cells which drives macrophage polarization and may contribute to gastric carcinogenesis.

## Material and methods

### Cells and cultures

Human immortalized gastric epithelium GES-1 and human monocyte THP-1 cell lines were routinely cultured in RPMI-1640 containing 10% fetal bovine serum (FBS) and 1% penicillin-streptomycin, at 37°C in a humidified incubator under 5% CO2. For the induction of THP-1-derived M0 macrophages, 100 ng/mL of phorbol 12-myristate 13-acetate (PMA) was added to THP-1 cells in RPMI-1640 for 24 h. Cultures were checked for mycoplasma on a regular basis. Peripheral blood mononuclear cells (PBMCs) were isolated using Ficoll (Amersham, Uppsala, Sweden) gradient density centrifugation [28]. The cells were suspended in 2 mL of IMDM media supplemented with Ultraglutamine and then placed in a T25 flask at a density of 1*10^6 cells per square centimeter. The cells were left to adhere for a duration of 60 min at a temperature of 37°C, after which the cells were subjected to two washes with PBS in order to eliminate any cells that were not adhered to the surface. Cells were subsequently cultured in IMDM containing L-glutamine, 10% FCS, 1% penicillin-streptomycin, and 50 ng/ml GM-CSF (Sigma-Aldrich).

### Stomach organoids cultures

Stomach organoids were cultured as described previously [29,30]. Briefly, both antrum and corpus biopsies were obtained from patients in the previously described Proregal cohort [31]. Gastric biopsies were washed, minced and digested using collagenase type IA (Sigma-Aldrich) to obtain a single cell suspension. Subsequently, matrigel (Corning, New York, United States) was inoculated with cells. Cells were maintained in either expansion or differentiation medium containing: Wnt3A conditioned medium (expansion medium only), Noggin conditioned medium, R-Spondin conditioned medium, FGF10 (Peprotech, Londen, United Kingdom), EGF (Peprotech), B27 (Invitrogen), Gastrin (Sigma-Aldrich), TGF-βi (A-83-01, Bristol, United Kingdom), Nicotinamide (Sigma-Aldrich, only expansion medium) and RHOKi (Y-27632, Sigma-Aldrich, only during initiation). For stimulations, Matrigel was first disrupted mechanically, followed by a gentle washing to remove as much Matrigel as possible without damaging the structure of the organoids. The organoid suspension was then divided equally across 24 well plates and stimulated with control medium or *H. pylori*.

### Flow cytometry

Using flow cytometry, the membrane expression of CD163, CD209, and CD80 was assessed in order to study macrophage polarization following stimulation. Thermo Fisher's CD163 (PE, cat. no. 12-1639-41), CD209 (APC, cat. no. 17-2099-41), and CD80 (FITC, cat. no. 11-0809-41) antibodies were diluted to 1:100, added to THP-1-derived macrophages for 30 min, and after washing with phosphate buffered saline (PBS) samples were analyzed by MACSQuant Flow Cytometer (Miltenyi Biotec, Gladbach, Germany). FlowJo v10 was used for data analysis (BD Biosciences).

### IL-6 measurement

To investigate the stimulation-induced IL-6 production in THP-1-derived macrophages and GES-1 cell lines, cells were plated at 10^6^/cells/well in 6-wells plates. Following a 24-hour incubation period, medium was refreshed and cells were stimulated with recombinant human IL6 (50 ng/mL; InvivoGen, San Diego, CA) or 10^6^ colony-forming units (CFU) of heat-killed *H pylori* (strain ATCC-43504 [cagA+, vacA(s1/m1), iceA+, and babA2+]; Manassas,VA). IL-6 production in supernatants was determined at 6, 24, 48, 72, 96, and 120 h of stimulation by enzyme linked immunosorbent assay (ELISA) (eBioscience, San Diego, CA), as per manufacturer's instructions. In short, the capture antibody was incubated overnight at 4°Celsius in an immunosorbent 96-well plate, after which each sample was added in duplicate, the detection antibody was added to bind with IL-6 from samples, and avidin-HRP was added to bind with the avidin-coated detection antibody. Tetramethylbenzidine (TMB) was added and the reaction was stopped after 30 min by addition of 2N H_2_SO_4_. Plates were read at 450 nm on a microplate reader (Infinite® M Nano, TECAN). Alternatively, for serum samples a non-competitive (sandwich) chemiluminescent immunoassay, the Roche Elecsys IL6 test was used. 18 µL of sample was first incubated with IL6-specific antibodies, and hen with IL6-specific antibodies that have been labeled with ruthenium complexes to create a sandwich complex. Complexes are then magnetically trapped, and the magnetic charge causes a chemiluminescent emission that is proportional to the amount of IL6 present, with a measurement range of 1.5–5000 pg/mL, and a limit of quantitation (LOQ) is 2.5 pg/mL.

### Quantitative real-time reverse-transcription polymerase chain reaction (qPCR)

Total cellular RNA was extracted and quantified using Macherey-Nagel NucleoSpin RNA II kit (Bioke, Leiden, The Netherlands) and Nanodrop ND-1000, respectively. Then, mRNA was transformed into cDNA using Primescript^TM^ RT Master Mix kit (Takara Bio, Saint-Germain-en-Laye, France) according to manufacturer's instructions and stored at -20°C. Real-time PCR was performed in a thermal cycler (GeneAmp PCR system 9700; Thermo Fisher) using SYBRGreen-based real-time PCR (Applied Biosystems). **Table S1** contains a list of each quantitative reverse-transcription PCR primer pairs. The annealing temperature for all primer combinations was 58 degrees Celsius. The ΔΔCT method was used to quantify the relative gene expression of each cytokine in each subgroup.

### Bioinformatic analysis

To investigate the increase in *IL-6* expression in samples related to *H. pylori* infection or stimulation, we performed data analysis using the DESeq2 Package from the R (version 4.0.2). We utilized publicly available gene expression datasets, including GSE186902, GSE162056, GSE25146, GSE230869, GSE27411, and GSE231337.

Additionally, we examined TCGA-GTEx data and perused individual RNA sequencing datasets (GSE191275) to verify the upregulation of *IL-6* expression in gastric cancer samples. We conducted the analysis using the R to assess the differential expression of *IL-6* in these datasets.

Furthermore, we utilized the TCGA dataset to analyze correlations between *IL-6* expression in gastric cancer patients with survival outcomes. Survival analysis was performed using GEPIA (http://gepia.cancer-pku.cn/), leveraging the TCGA gastric cancer data.

TCGA gastric cancer dataset was also used to explore the correlation between *IL-6* expression and specific macrophage markers, namely the M1 marker CD80 and the M2 marker CD163. We conducted correlation analysis using GEPIA to determine the relationship between *IL-6* expression levels and the expression of *CD80* and *CD163*.

Tumor Immune Infiltration was investigated using the CIBERSORT algorithm via the "CIBERSORT" R package (CIBERSORT R script v1.03; http://cibersort.stanford.edu/) to determine the relative abundance of 22 categories of tumor-infiltrating immune cells and non-immune cells in the gastric cancer tumor microenvironment using the TCGA and GTEx datasets.

### Single cell RNAseq data analysis

We analyzed a total of 15 samples from 10 patients, including three non-atrophic gastritis (NAG), three chronic atrophic gastritis (CAG), six IM, and two GC samples. The data from two collections of raw scRNA-seq data were used: 1) gene expression omnibus (GEO) accession number GSE134520, consisting of 3 NAG, 3 CAG, and 6 IM samples; 2) accession number phs001818.v2 of the database of Genotypes and Phenotypes (dbGaP), containing three GC samples. These samples represented the entire spectrum of disease, from gastritis to GC. The outputs from the expression count meta-matrix were converted into data objects using R package ‘Seurat’. Individual loaded Seurat data objects were merged iteratively using the ‘FindIntegrationAnchors’ function. Cells that expressed fewer than 200 genes, had more mitochondrial genes than 20%, or had a number of UMIs were excluded. With a default scale parameter of 10,000, the 'NormalizeData' function was used to normalize the data to log scale. Cell types were identified using markers defined in the original reports from which the data derived. Each marker (panel) is listed in Supplementary datafile **Table S2**.

The default settings of the R package ‘CellChat’ were used to analyze cellular connections.

### Cell viability assay

3-[4,5-dimethylthiazol-2-yl]-2,5 diphenyl tetrazolium bromide (MTT) assay was performed to quantify viable cell numbers. After IL-6 stimulation of GES-1 cells for 0, 3, and 24 h, MTT was added to a final concentration of 0.5 mg/ml, and plates were incubated for 3 h at 37°C. After that, 100 µl di-methyl-sulfoxide (DMSO) was added to each test well. Prior to analyzing the absorbance at 540 nm, the plate was incubated for 10 min at 37°C and subsequently shaken for one minute to dissolve crystals.

### Cell migration assay

To assess cellular migration, we performed wound-healing assays. To this end, GES-1 cells were plated at 10^5^ cells/ml and cultured until 50% confluence in a 6-well plate. The cells were then stimulated for 24 h with recombinant human IL6 (50 ng/mL; InvivoGen, San Diego, CA) or 10^6^ CFU of heat-killed *H pylori* (strain ATCC-43504, Manassas,VA). A straight scratch line was then created using a sterile 1000 µl pipette tip. Dishes were carefully rinsed to eliminate detached cells, and fresh culture medium with stimulator was added. At 0, 6, 12, and 24 h, cells that migrated into the scratch line were photographed. Image J was used to analyze the migrated distance in these images, and migration was presented as migration speed.

### Co-culture experiments

The GES-1 cell line and THP-1-derived macrophages were co-cultured using a cell culture insert (Corning, NY, USA) with a 0.4-μm porous membrane to separate the upper and lower chambers [32]. Inserts were coated with 5 g/cm^2^ of bovine collagen type I. The coating was permitted to set for 2 h at 37℃. Inserts were then washed with 1x PBS three times. THP-1 monocytes (5 × 10^5^ cells/ml) were seeded onto an plate, stimulated to differentiate into macrophages by the addition of 100 ng/ml PMA (Sigma Chemical) for 48 h. The GES-1 cells were placed in the upper chamber at a density of 2.5 × 10^5^ cells/ml for 24 h. Differentiated THP-1 cells were harvested by Trypsin dissociation and counted. 1 × 10^5^ cells were seeded on the bottom of a transwell insert containing a confluent GES-1 monolayer in a 50 µl droplet. Inserts were incubated for 2 h and were reversed. The inserts with the THP-1-derived macrophages and GES-1 cells separated by the membrane were then placed directly in a six-well plates, and the resulting co-culture systems were incubated for 48 h with or without 10^6^ colony-forming units of heat-killed *H pylori*. Medium was collected from the apical (insert) and basolateral side (lower well).

### Patients

48 patients at The First Affiliated Hospital of Zhengzhou University with histologically proven stomach cancer participated in this study from February 2023 to August 2023. The study was approved by the hospital review board after receiving the informed consent of every patient. All patients underwent C13 breath testing to determine their *H. pylori* infection status, and serum samples were taken to assess their IL-6 levels via the Roche Elecsys IL6 test. We procured both tumor and adjacent tissues of 7 of these patients, followed by sectioning and subsequent application of immunohistochemical staining analysis.

### Immunohistochemistry

In brief, xylene was used twice to deparaffinize 4 µm sections before they were rehydrated using graded ethanol solutions of 100% ethanol twice, 96% solution ethanol, and 70% solution ethanol. Slides were washed once with tap water after being rinsed several times with fresh deionized water. In order to achieve heat-induced epitope retrieval, 10 mM sodium citrate buffer (pH 6.0) was used for 15 min. Slides were gently chilled for 45 min after epitope retrieval, then three times for 5 min in PBS. PBS/3% H2O2 solution was used to inhibit endogenous peroxides for 10 min at room temperature (RT). Slides were cleaned with PBS before being blocked with 10% normal goat serum in PBS for an hour at room temperature. The CD163 primary antibody (CD163 (D6U1J) Rabbit mAb, #93498, Cell signaling) was then added, and the mixture was incubated at 4°C overnight. PBS was used to wash the slides. As a secondary antibody, rabbit envision (DAKO) was added and incubated for 30 min at room temperature. A Tris/HCL solution (pH 7.6) containing 0.03% H2O2 and 0.5 mg/ml diamino-benzidine (DAB) was used for visualization, which was incubated for 10 min. Slides were mounted with Pertex and a coverglass, counterstained with hematoxylin, rinsed with tap water, dehydrated with a 70% solution of ethanol, then a 96% solution of ethanol, twice in 100% ethanol, and finally twice in xylene. Stainings were scored based in intensity and proportion of positive cells using H-scoring ([(0 x % negative cells) + (1 x % weak positive cells) + (2 x % moderate positive cells) + (3 x % strong positive cells).

## Results

### IL-6 is highly expressed in GC tissues and drives oncogenic characteristics

**Fig. 1:**
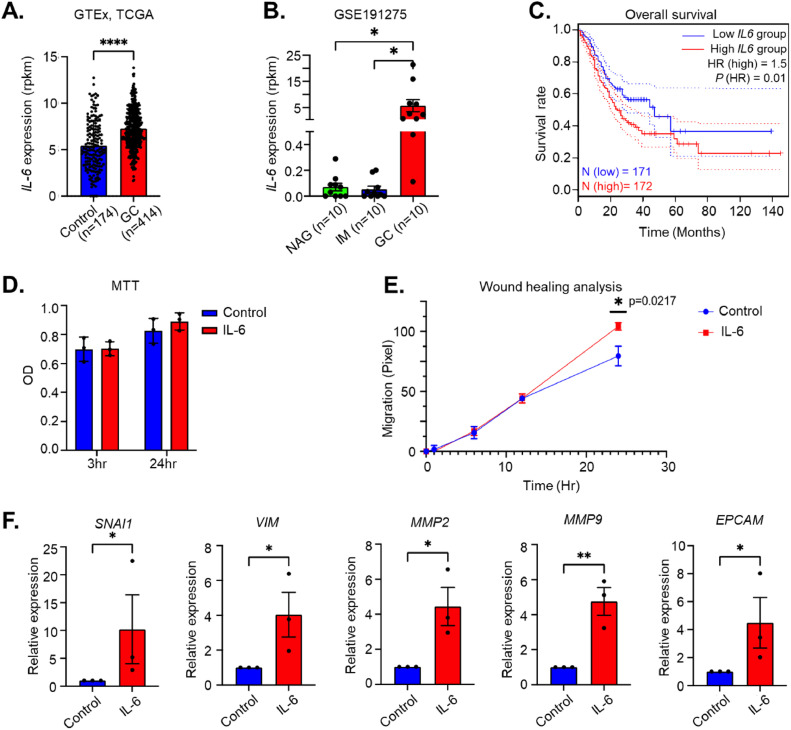
IL6 levels are increased in gastric cancer tissue. A. Gastric cancer tumor samples (GC; TCGA-STAD) present a significantly higher *IL-6* mRNA expression than normal stomach samples (Control; GTE-x dataset). B. Gastric cancer (GC) samples show a significantly higher *IL-6* expression than intestinal metaplasia (IM) or non-atrophic gastritis (NAG) samples (GSE191275 dataset). C. Patients with high *IL-6* mRNA levels in their GC tissues show a significantly reduced overall survival time (TCGA dataset). D. MTT assay indicates that IL-6-stimulated GES-1 cells do not exhibit an increased proliferation ability. E. IL-6 treatment of GES-1 cells significantly enhances their migratory capacity in wound healing assays. F. GES1 cells stimulated with IL-6 present an increase in relative mRNA expression of the gastric cancer hallmarks *SNAI1, VIM, MMP2, MMP9* and *EPCAM*, respectively. Mean ± standard deviation of three independent experiments is shown. *P<0.05, **P<0.01, ***P<0.001.

We first compared the *IL-6* mRNA expression in publicly available datasets from healthy stomach samples (*n=*174, GTEx) and tumor samples (*n=*414, TCGA-stomach adenocarcinoma) and showed that *IL-6* expression was significantly enhanced in tumor tissues (*P* ≤ 0.0001) (Fig. 1A). This was further substantiated through analysis of another publicly available dataset (GSE191275), which indicates a significantly higher *IL-6* expression in GC tissues (*n=*10) compared to non-atrophic gastritis (NAG, *n=*10) and intestinal metaplasia (IM, *n=*10) (*P* ≤ 0.0001) (Fig. 1B). In addition, high *IL-6* expression (above the 50th percentile) was associated with significantly decreased survival rates in GC patients (TCGA data, Fig. 1C).

To investigate whether IL-6 may directly contribute to gastric carcinogenesis, we employed an *in vitro* model using non-transformed gastric epithelial cells (GES-1). While IL-6 did not affect their viability, as indicated by the MTT assay (Fig. 1D), stimulation of these cells with IL-6 significantly enhanced their migration capacity, as demonstrated by the wound healing assay **(**Fig. 1E). These results suggest that IL-6 predominantly affects cell migration rather than proliferation.. In addition, expression of genes involved in epithelial to mesenchymal transition (EMT: *SNAI1*, encoding Snail and *VIM* encoding Vimentin) were significantly increased after 48 h of IL-6 stimulation, as were genes involved in extracellular matrix remodeling (matrix metalloproteinases *MMP2, MMP9*) and the cancer marker *EPCAM* [33] (Fig. 1F**)**. Thus, IL6 levels are enhanced in GC tissue, and drive hallmarks of malignant potential.

### IL-6 mediates a cross-talk involving macrophages and tumor cells in the tumor microenvironment

**Fig. 2:**
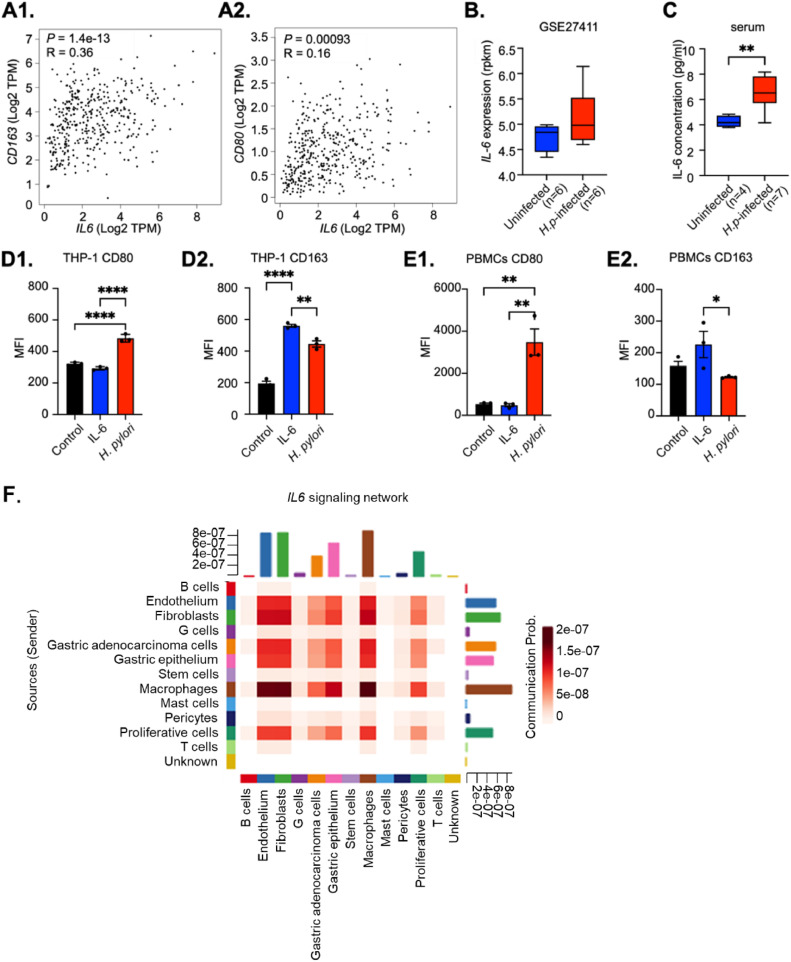
IL-6 drives M2 macrophage polarization. A. In gastric cancer samples from the TCGA dataset, *IL-6* is significantly correlated with the M2 macrophage gene *CD163*, and to a lesser extent with the M1 Macrophage gene *CD80*. B. Analysis of publicly available RNA sequencing dataset illustrates that *IL-6* mRNA levels are elevated in gastric tissue from *Helicobacter pylori* (*H.p*)-infected individuals antrum. C. Serum levels of IL-6 protein are higher in *Helicobacter pylori* (*H.p*)-infected individuals compared to non-infected controls. D, E. Flow cytometric analysis of expression of CD163 and CD80 on THP-1 derived macrophages (D) or primary monocyte-derived macrophages (E) stimulated with IL-6 or *H. pylori*. Results are presented as mean fluorescence intensity of the cell populations. Mean ± standard deviation of three independent experiments is shown. *P<0.05, **P<0.01, ***P<0.001. F. Heat map generated using the CellChat function illustrating the sources and targets of *IL-6* signaling and the total outgoing and incoming interactions of IL-6 scores.

IL-6 has been thought to play a key role in the cross-talk between different cell lineages within the tumor microenvironment. To investigate the cell types involved in GC, we utilized the Cell Chat package to determine the inferred intercellular communication network for IL-6 signaling (**Supplementary Fig. S1**). The ensuing circle plot reveals that a strong association exists through IL-6 signaling between macrophages and the gastric epithelium as well as adenocarcinoma cells. Indeed, the main source of IL-6 signaling in this setting are macrophages (see heat map, Fig. 2F), which in turn show the largest association with gastric epithelial and adenocarcinoma cells. To investigate the importance of this finding, we analyzed the distribution of inferred immune cell subsets in gastric cancer using the TCGA dataset. Our findings reveal that in particular M2 macrophages are the second largest infiltrating immune cell subset among the 22 immune cell types evaluated, and significantly increased in gastric cancer tissues (**Supplementary Fig. S2A**). To validate these observations, we analyzed single cell RNAseq data sets from three NAG, three CAG, six IM and three GC samples **(Supplementary Fig. S2B**), and observed a consistent increase in the percentage of macrophages in GC samples compared to pre-malignant lesions. In particular the percentage of CD163+ M2 macrophages increased in gastric cancer samples (**Supplementary Fig. S2C**), showing that M2 macrophages are an integral part of the gastric microenvironment during carcinogenesis. Furthermore, immunohistochemical analysis of GC and adjacent paracancer tissues showed that almost 30% of tumor tissues exhibited high expression of CD163, while none of the adjacent paracancer tissues showed a high H score. Conversely, 40% of adjacent paracancer tissues vs 30% of cancer tissues demonstrated a low H score (**Supplementary Fig. S2D**). **Supplementary Fig. S1** further indicates that IL-6 signaling displays both autocrine loops as well as reciprocal paracrine loops between the diverse cell lineages within this microenvironment.

### *H. pylori* and IL-6-mediated macrophage polarization

To further explore the relationship between IL-6 and M2 macrophage polarization we again queried the TCGA gastric tumor dataset. We observed a significant positive correlation between *IL-6* expression and the M2 macrophage marker *CD163* (R=0.36, *P*=1.4e-13), while the correlation between *IL-6* expression and the M1 macrophage marker *CD80* was less strong (R=0.16, *P*=0.00093) (**Fig. 2A1, A2**). One of the first triggers contributing to IL-6 production and macrophage polarization in gastric carcinogenesis may be infection with *H. pylori*, as higher *IL-6* mRNA levels are present in gastric mucosa (*n=*6 vs *n=* 6, *P* = 0.0891, Fig. 2B) as well as serum from *H. pylori*-infected healthy individuals (*n=*7 vs *n=* 4, *P* = 0.01, Fig. 2C) and cancer patients (*n=*24 vs *n=*24, *P* =0.3847 **Supplementary Fig. S3**) compared to uninfected controls. To directly investigate whether *H. pylori* and/or IL-6 affect macrophage polarization, we conducted stimulation experiments on THP-1 cells, a monocytic cell line which can be induced to differentiate towards the macrophage lineage through PMA stimulation [34]. Somewhat unexpectedly, stimulation of THP-1 derived monocytes with *H. pylori* resulted in a significantly enhanced expression of the M1 marker CD80 (**Fig. 2D1**), with limited effect on CD163 expression (**Fig. 2D2**). Conversely, IL-6 enhanced expression of CD163 on THP-1-derived macrophages, but did not affect CD80 expression on these cells (**Fig. 2D1, D2**). These experiments were verified using peripheral blood-derived macrophages, which showed similar results (**Fig. 2E1, E2**).Thus, acute *H. pylori* infection may skew macrophages to an M1 phenotype, while IL-6 (potentially as a result of long term infection) polarizes macrophages towards the M2 lineage. This dual effect highlights the intricate relationship between the duration of H. pylori infection and cytokine levels.

### IL-6 enhances its own production in gastric cells and macrophages

**Fig. 3:**
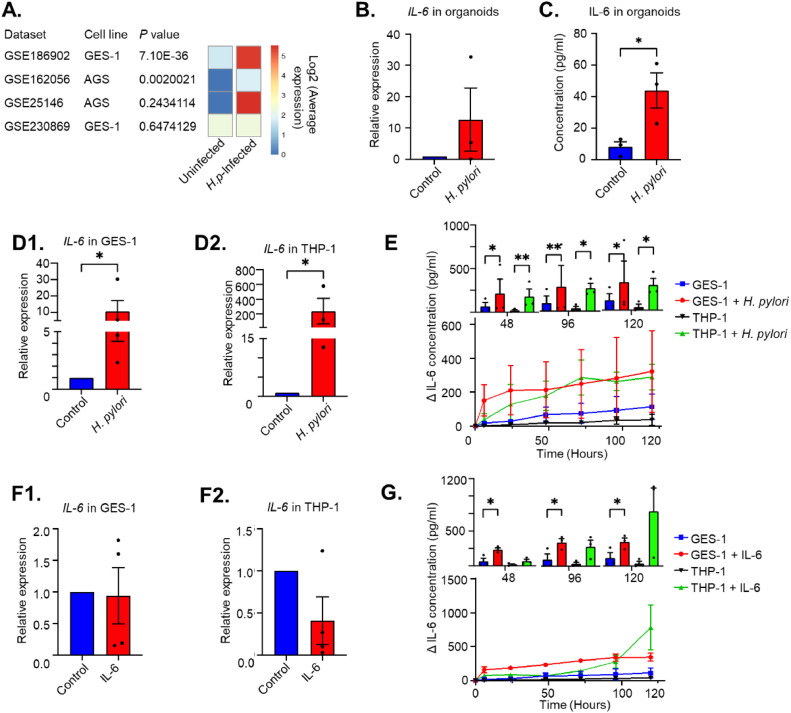
*H. pylori* as well as IL-6 enhance IL-6 expression in macrophages and epithelial cells. A. Publicly available RNA datasets were searched for gastric epithelium cell lines stimulated with *H. pylori* and queried for *IL-6* expression. Four datasets were found, all showing an increase in IL-6 expression upon *H. pylori* stimulation of gastric cell lines. B. Gastric organoids were stimulated with *H. pylori* and IL-6 mRNA levels were determined by qPCR. C. Gastric organoids stimulated with *H. pylori* demonstrate a significant increase in IL-6 release as determined by ELISA. D. *IL-6* mRNA expression in GES-1 (D1) and THP-1 (D2) cell-lines after stimulation with heat-killed *H. pylori*. E. IL-6 protein concentration in medium collected from GES-1 and THP1cell lines after stimulation with heat-killed *H. pylori*. Lower panels show time curve of IL-6 levels, upper panels show comparison per time point of collection. F. *IL-6* expression in GES-1 (F1) and THP-1 (F2) cell-lines after stimulation with human recombinant IL6. G. IL-6 concentration in medium collected from GES-1 and THP-1 cell lines after stimulation with human recombinant IL6. Lower panels show time curve of IL-6 levels after subtraction of added recombinant protein (Δ), upper panels show comparison per time point of collection. Mean ± standard deviation of three independent experiments is shown. *P<0.05, **P<0.01, ***P<0.001.

To investigate the cross-talk between different cell populations in IL-6 production in more detail, we employed GES-1 epithelial cells as well as THP-1 cells. First, we verified that these models were able to generate IL-6. Consistent with data from publicly available RNA sequencing data-sets (Fig. 3A) and primary gastric organoids (Fig. 3B**,** C), stimulation of GES-1 cells with *H. pylori* results in a significant increase in *IL-6* mRNA production within 24h (**Fig. 3D1**), with secretion of IL-6 showing a rapid start and subsequent plateau (Fig. 3E). Differentiated THP1 cells also significantly upregulate their *IL-6* mRNA production upon *H. pylori* infection (**Fig. 3D2**), with a more gradual IL-6 protein release, which overtakes GES-1-produced IL-6 levels after 96 h (Fig. 3E).

Having confirmed the IL-6 production ability of GES-1 and THP-1 cells, we examined whether they exhibit an autocrine activation pattern, as suggested earlier (**Fig. Supplementary S1**). Although stimulation with recombinant IL-6 did not significantly affect *IL-6* mRNA levels in GES-1 and THP-1 cells (**Fig. 3F1, F2**), Fig. 3G demonstrates that the medium obtained from both cell lines contained IL-6 levels that exceeded the added recombinant protein levels. This suggests that IL-6 induces its own protein expression in these cells, highlighting an autocrine activation mechanism.

### *H. pylori*-stimulated macrophages further induce IL-6 production in gastric cells

**Fig. 4:**
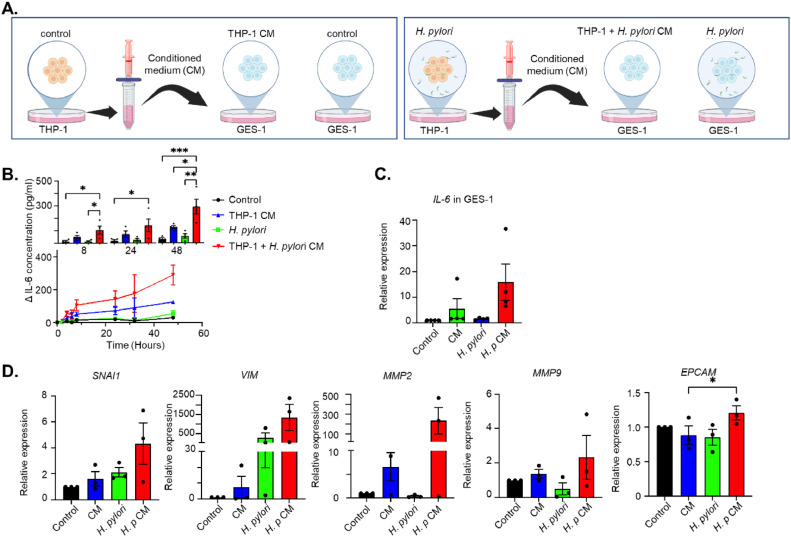
*H. pylori* stimulation of THP-1-derived macrophages causes release of factors triggering IL-6 production in GES-1 cells. A. Schematic representation of the experiment. Conditioned media (CM) was collected from THP-1-derived macrophages and filtered to remove cell debris and *H. pylori*. GES-1 cells were subsequently stimulated with these CM, with control wells stimulated with *H. pylori* directly or medium control. B, C. IL-6 protein concentration in medium collected from GES-1 cells after stimulation with CM from THP1 cells (treated or non-treated with *H. pylori*) or *H. pylori.* heat-killed *H. pylori*. Lower panels show time curve of IL-6 levels, upper panels show comparison per time point of collection. C. IL-6 mRNA expression in GES-1 stimulated as shown in panel A D. Relative mRNA expression of the gastric cancer hallmarks *SNAI1, VIM, MMP2, MMP9* and *EPCAM*, respectively, in GES-1 cells stimulated as depicted in panel A. Mean ± standard deviation of three independent experiments is shown. *P<0.05, **P<0.01, ***P<0.001.

**Fig. 5:**
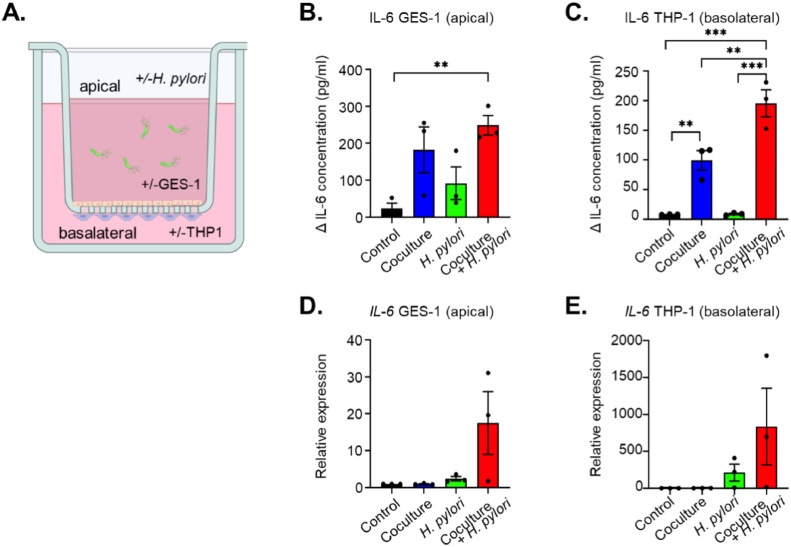
Co-culture of THP1 and GES-1 cells in the presence of *H. pylori* synergistically enhances IL-6 production. A. Schematic presentation of co-culture set-up. Medium and cells were collected from co-cultures of THP-1-derived macrophages and GES-1 cells (with and without addition *H. pylori*) of the basolateral side (THP1) and apical side (GES-1), and compared to mono-cultures of THP-1 or GES-1 in the absence or presence of *H. pylori*, respectively. B. IL-6 concentration in medium obtained from GES-1 cells. C. IL-6 concentration in medium obtained from from THP-1 cells. D. Quantification of *IL6* mRNA levels in GES-1 cells stimulated as described in panel A. E. Quantification of *IL-6* mRNA in THP-1-derived macrophages stimulated as described in panel A. Mean ± standard deviation of three independent experiments is shown. *P<0.05, **P<0.01, ***P<0.001

To investigate the role of the IL-6 feedback loop in the cross-talk between different cell populations, conditioned medium was collected from *H. pylori*-stimulated or non-stimulated THP-1-derived macrophages. Subsequently, we treated GES-1 cell lines with these conditioned media, using culture media with or without *H. pylori* as controls (Fig. 4A). Following stimulation of GES-1 cells with THP-1- conditioned media, we observed a continuous increase in the secretion of IL-6. Intriguingly, conditioned medium from *H. pylori*-stimulated macrophages (which no longer contains *H. pylori*), induced a more pronounced increase in IL-6 levels compared to direct stimulation of GES-1 cells with *H. pylori* or conditioned medium from unstimulated THP-1 cells (Fig. 4B). Consistent with these findings, *IL-6* mRNA levels also showed the highest elevation after stimulation with the *H. pylori*-stimulated THP-1 conditioned media (Fig. 4C). Moreover, GES-1 cell lines stimulated with the conditioned medium from *H. pylori*-stimulated macrophages exhibited the highest increase in expression of *EPCAM, SNAI1, VIM, MMP2,* and *MMP9* mRNA compared to control conditions (Fig. 4D). To further validate the cross-talk observed between GES-1 cells and THP-1-derived macrophages mediated by the IL-6 feedback loop, we co-cultured these two cell types in a transwell culture system, allowing subsequent harvesting of cells and culture media separately from basolateral and apical sides of the insert (Fig. 5A). Again, co-culture of these cells resulted in an enhanced release of IL-6 from GES-1 (Fig. 5B) and THP-1-derived macrophages (Fig. 5C). In addition, co-culture with *H. pylori* resulted in a synergistic effect, increasing levels of IL-6 more than either *H. pylori* alone or co-culture without the presence of *H. pylori*, in particular for THP-1 cells (Fig. 5B and C). This was also reflected in the *IL6* mRNA levels obtained from either GES-1 cells (Fig. 5D) or THP-1-derived macrophages (Fig. 5E). These findings suggest that stomach epithelial cells and macrophages increase each other's IL-6 levels, and that this effect is synergistically enhanced when either one or both cell lineages are primed with *H. pylori*.

### IL-6 induces a positive feedback loop between GES1 and THP1-derived macrophages

**Fig. 6:**
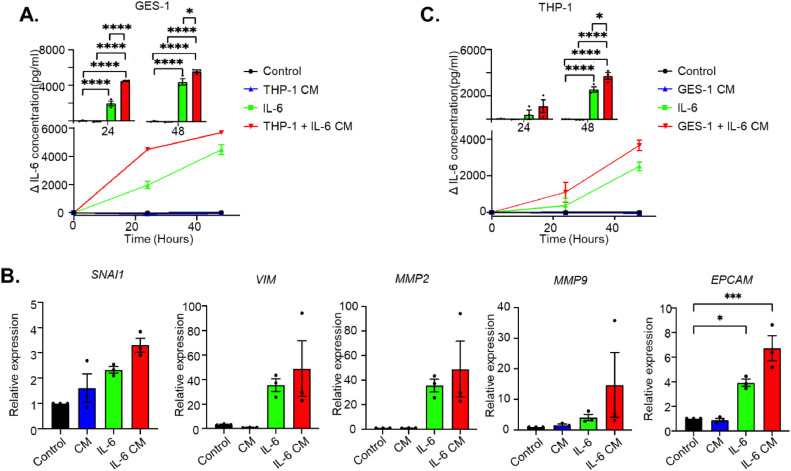
IL-6 participates in a positive feedback loop between GES-1 cells and THP-1-derived macrophages. A. IL-6 protein concentration in medium collected from GES-1 cells after stimulation with CM from THP1 cells (treated or non-treated with human recombinant IL-6) or human recombinant IL-6 directly*.* Lower panels show time curve of IL-6 levels after subtraction of added recombinant protein (Δ), upper panels show comparison per time point of collection. B. Relative mRNA expression of the gastric cancer hallmarks *SNAI1, VIM, MMP2, MMP9* and *EPCAM*, respectively, in GES-1 cells stimulated as described for A. C. IL-6 protein concentration in medium collected from THP-1 cells after stimulation with CM from GES-1 cells (treated or non-treated with human recombinant IL-6) or human recombinant IL-6 directly*.* Lower panels show time curve of IL-6 levels after subtraction of added recombinant protein (Δ), upper panels show comparison per time point of collection. Mean ± standard deviation of three independent experiments is shown. *P<0.05, **P<0.01, ***P<0.001.

Continuing our investigation into the IL-6 feedback loop and its impact on cross-talk between different cell populations in gastric carcinogenesis, we performed additional experiments as in Fig. 5A, but priming THP-1 cells with IL-6 instead of *H. pylori*. Fig. 6A shows that GES-1 stimulated with IL-6 start increasing their IL-6 secretion (see also Fig. 3G). However, IL-6 secretion is even further enhanced when GES-1 cells are treated with conditioned medium from THP-1-derived macrophages who have previously been primed by IL-6 (Fig. 6A). Conditioned medium of IL-6 primed macrophages also induced the highest expression level of EMT-associated genes and cancer hallmarks *SMAI1, VIM, MMP2, MMP9* and *EPCAM* in GES-1 cells (Fig. 6B). We subsequently performed the reverse experiment, i.e. priming GES-1 cells with IL-6 and treating THP-1 macrophages with conditioned medium from GES-1 cells. As for gastric epithelium cells, IL-6 production in macrophages is significantly enhanced when treated with conditioned medium from IL-6-primed GES-1 cells (Fig. 6C). Together, these findings indicate the existence of an IL-6 feedback loop which can amplify the IL-6 signaling cascade in both gastric epithelial cells (GES-1) and macrophages (THP-1), enhancing hallmarks of progression of gastric carcinogenesis.

## Discussion

The mechanism behind *H. pylori*-associated inflammation and GC development remains incompletely understood, and identifying factors that could contribute to these processes remains important. A previous meta-analysis identified IL-6 as a potential key factor in this process [11], making it a subject for further investigation. We aimed to explore the role of IL-6 in *H. pylori*-associated gastric cancer and show that macrophages and epithelial cells demonstrate both autocrine and paracrine IL-6 induction, which is enhanced by *H. pylori*.

Overall, our data suggest that IL-6 can enhance its own levels in the gastric microenvironment, which can contribute to the development of carcinogenic properties. This is in line with previous studies showing that IL-6 mediates cellular cross-talk in the microenvironment in other cancer types [35]. A self-stimulatory role for IL-6 has been shown for instance for fibroblasts in vocal fold leukoplakia [36] and for non-small cell lung carcinoma (NSCLC) [37]. The action of IL-6 is regulated by an intracellular network of effectors, including its receptor (IL-6R and gp130), engagement of which results in phosphorylation of the Janus kinase (JAK)2 and its downstream effector signal transducer and activator of transcription (STAT)3. Activated STAT3 translocates to the nucleus, where it initiates the transcription of a series of genes responsible for cell growth, apoptosis inhibition, and cell cycle progression[38]. Accumulation of unphosphorylated STAT3 in response to IL-6 can subsequently result in autocrine expression of the *IL-6* gene [39], while nuclear STAT3 was shown to bind to the *IL6* promotor [40]. However, other intermediate signaling events may also contribute to the IL-6 induced positive feedback loop. In addition, *H. pylori* is also a known activator of STAT3, which in part may account for its induction of IL-6 production [41]. However, during inflammation and tissue repair, the action of IL-6 is strictly controlled by negative regulators such as the suppressor of cytokine signaling (SOCS) family members [42]. *H. pylori* has been found to induce hypermethylation of SOCS1 [43], which can reverse the suppression of *IL-6*. Additionally, IL-6 itself has been found to increase hypermethylation of SOCS3 [44], all of which may contribute to a positive feedback loop in the *H. pylori*-infected tumor microenvironment.

While autocrine IL6-JAK-STAT3 signaling has been shown before in GC cells [45], the role of this autocrine loop in the cross-talk between different cells of the microenvironment remained unclear. Previous studies showed that IL-6 derived from mesenchymal cells can drive polarization of M2 macrophages in GC [46,47]. Our data suggest that IL-6 produced in the local microenvironment may also contribute to M2 polarization, as well as subsequent IL-6 production by these cells. Such a mechanisms appears to take place in lung cancer, where conditioned medium from Lewis lung cancer cells was shown to drive M2 polarization and IL-6 production in PBMCs [48].

Our data indicate that whilst IL-6 drives M2 polarization of macrophages, *H. pylori* may affect mainly M1 polarization, which is in line with previous results [49]. This would fit in an hypothesis where initial infection with *H. pylori* in the (then) healthy stomach induces an inflammatory phenotype associated with M1 polarization, whereas once a chronic infection has established, ongoing auto/paracrine IL-6 production induces a switch towards M2 polarization and might promote gastric carcinogenesis.

## Conclusion

This study confirms that IL-6 within the tumor microenvironment plays a crucial role in the intercellular communication. We demonstrate that IL-6 acts in a positive feed-back loop in both autocrine and paracrine fashion between macrophages and gastric epithelial cells, which can be further enhanced by *H. pylori*. These data provide a step forward in our understanding of the role of IL-6 in GC. Further research is required to elucidate the intracellular mechanisms involved.

## Funding

Authors of the First Affiliated Hospital of Zhengzhou University were funded through Funding for Scientific Research and Innovation Team (QNCXTD2023022).

## Data availability

All data generated or analyzed during this study can be downloaded from TCGA (https://portal.gdc.cancer.gov/), GTEx (https://www.gtexportal.org/home), and GEO datasets(https://www.ncbi.nlm.nih.gov/geo/).

## Ethics approval and consent to participate

Not applicable.

## Consent for publication

Not applicable.

## CRediT authorship contribution statement

**Bingting Yu:** Conceptualization, Data curation, Formal analysis, Methodology, Visualization, Writing – original draft. **Danny de Vos:** Data curation, Formal analysis, Methodology, Validation, Visualization, Writing – review & editing. **Xiaopei Guo:** Formal analysis, Methodology, Writing – review & editing. **SanFei Peng:** Data curation, Investigation, Writing – review & editing. **Wenjie Xie:** Data curation, Investigation, Writing – review & editing. **Maikel P. Peppelenbosch:** Project administration, Resources, Supervision, Writing – review & editing. **Yang Fu:** Project administration, Resources, Supervision, Writing – review & editing. **Gwenny M. Fuhler:** Conceptualization, Project administration, Resources, Supervision, Visualization, Writing – original draft.

## Declaration of competing interest

The authors declare that they have no known competing financial interests or personal relationships that could have appeared to influence the work reported in this paper.

## Appendix Supplementary materials

Image, application 1

Image, application 2
