# Supplementary material for: IL-6 facilitates cross-talk between epithelial cells and tumor- associated macrophages in Helicobacter pylori-linked gastric carcinogenesis
Source: Neoplasia. 2024 Feb 28;50:100981. doi: 10.1016/j.neo.2024.100981 (PMC10912637; doi:10.1016/j.neo.2024.100981)
Supplement: Supplementary file 2 [file mmc2.docx]

**
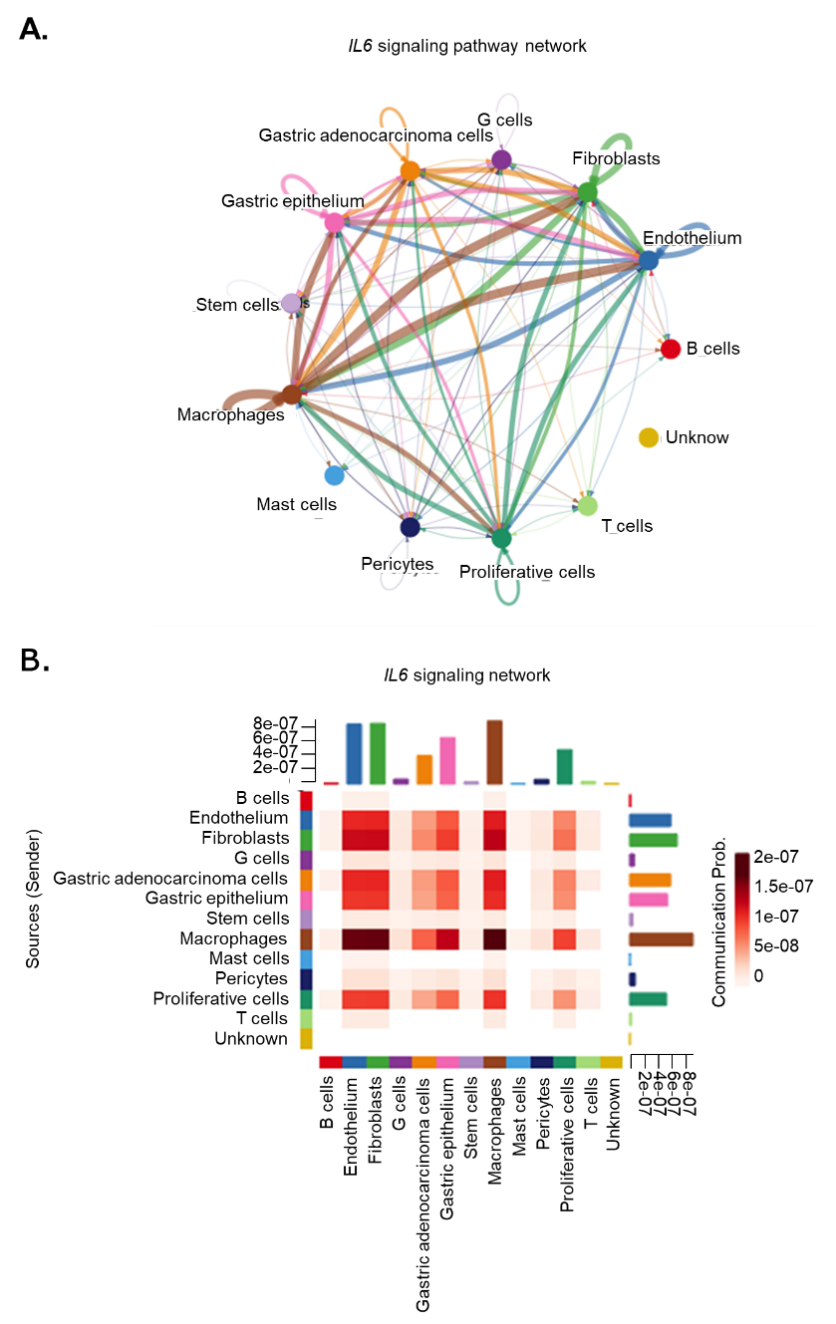
**

**Figure S1. Single cell data analysis indicates that *IL-6* mediates a cross-talk between different cell types in the gastric tumor microenvironment.** Circle plot analyzing the role of *IL-6* in the cell-cell communication between interacting cell groups within one environment points toward a cross-talk between macrophages, fibroblasts and tumor cells, with paracrine and autocrine *IL-6* signaling.


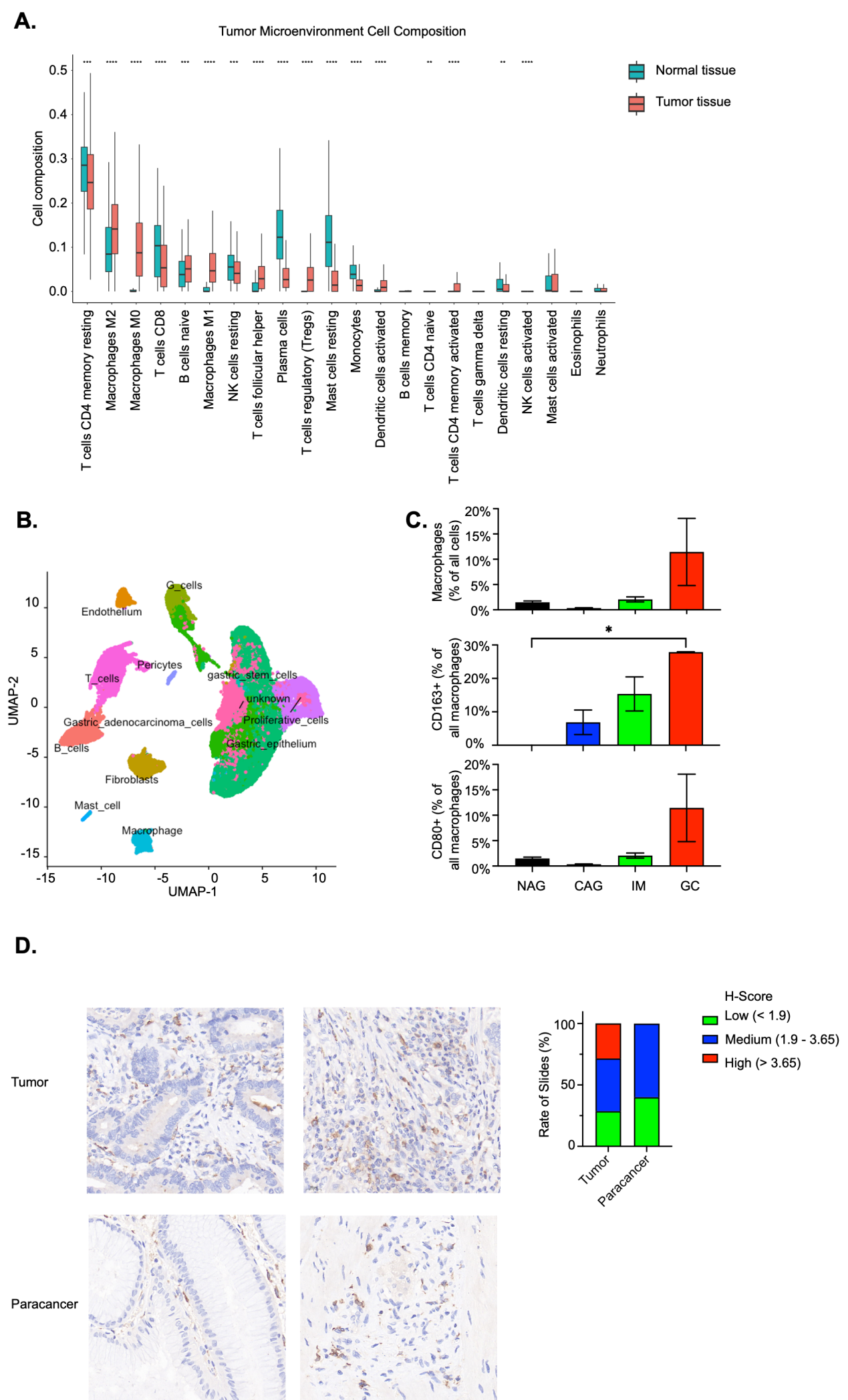


**Figure S2 Infiltration of macrophages, particularly M2 macrophages, in gastric cancer A.** Distribution of inferred immune cell subsets in gastric cancer tissues from TCGA dataset as determined with CIBERSORTx. **B.** UMAP plot of 48,964 cells derived from 15 gastric tissue samples, with cells color-coded according to their clustered cell type. Macrophages were defined by their expression of *CD14*, *CD68*, and *FCGRA* **C.** Upper panel: percentage of macrophages present in gastric mucosa at various stages of gastric carcinogenesis. Middle panel: percentage of macrophages in gastric mucosa expressing the M2 marker *CD163*. Lower panel: percentage of macrophages in the gastric mucosa expressing the M1 marker *CD80.* Mean ± standard deviation is shown, *P<0.05. **D.** Representative immunohistochemical staining examples of CD163 expression in tumor tissue (upper left panels) and adjacent paracancerous tissue (lower left panels), as well as quantification of the immunohistochemical H-index, categorized into low, medium and high expression (right panel).

**
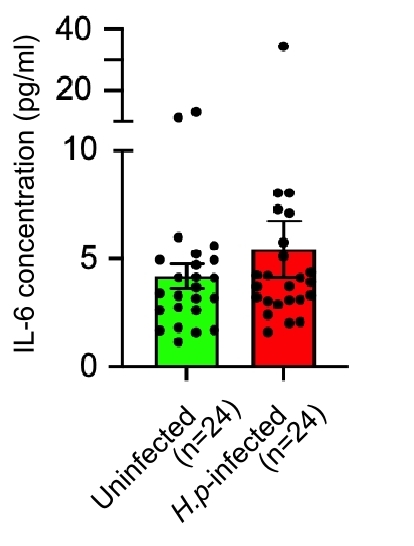
Figure S3**, IL-6 concentration in Serum of gastric cancer patient with/without H.pylori infection
